# CCN3 Facilitates Runx2 and Osterix Expression by Inhibiting miR-608 through PI3K/Akt Signaling in Osteoblasts

**DOI:** 10.3390/ijms20133300

**Published:** 2019-07-05

**Authors:** Po-Chun Chen, Ju-Fang Liu, Yi-Chin Fong, Yuan-Lin Huang, Chia-Chia Chao, Chih-Hsin Tang

**Affiliations:** 1 Central Laboratory, Shin-Kong Wu Ho-Su Memorial Hospital, Taipei 111, Taiwan; 2 Department of Sports Medicine, College of Health Care, China Medical University, Taichung 404, Taiwan; 3 Department of Orthopaedic Surgery, China Medical University Beigang Hospital, Beigang 651, Taiwan; 4 Department of Biotechnology, College of Health Science, Asia University, Taichung 413, Taiwan; 5 Department of Respiratory Therapy, Fu-Jen Catholic University, New Taipei City 242, Taiwan; 6 Department of Pharmacology, School of Medicine, China Medical University, Taichung 404, Taiwan; 7 Graduate Institute of Biomedical Science, China Medical University, Taichung 404, Taiwan; 8 Chinese Medicine Research Center, China Medical University, Taichung 404, Taiwan

**Keywords:** CCN3, osteoblasts, Runx2, osterix, miR-608

## Abstract

CCN3, otherwise known as the nephroblastoma overexpressed (NOV) protein, is a cysteine-rich protein that belongs to the CCN family and regulates several cellular functions. Osteoblasts are major bone-forming cells that undergo proliferation, mineralization, renewal, and repair during the bone formation process. We have previously reported that CCN3 increases bone morphogenetic protein 4 (BMP-4) production and bone mineralization in osteoblasts, although the role of CCN3 remains unclear with regard to osteogenic transcription factors (runt-related transcription factor 2 (Runx2) and osterix). Here, we used alizarin red-S and alkaline phosphatase staining to show that CCN3 enhances osteoblast differentiation. Stimulation of osteoblasts with CCN3 increases expression of osteogenic factors such as BMPs, Runx2, and osterix. Moreover, we found that the inhibition of miR-608 expression is involved in the effects of CCN3 and that incubation of osteoblasts with CCN3 promotes focal adhesion kinase (FAK) and Akt phosphorylation. Our results indicate that CCN3 promotes the expression of Runx2 and osterix in osteoblasts by inhibiting miR-608 expression via the FAK and Akt signaling pathways.

## 1. Introduction

Osteoblasts are major bone-forming cells that undergo proliferation, mineralization, renewal, and repair during the bone formation process [1,2]. Accumulating evidence indicates that agents capable of enhancing osteoblastic proliferation or increasing the differentiation of osteoblasts promote bone formation [3,4], thus justifying the approval of the bone formation compound teriparatide 1–34 for osteoporosis therapy [5].

Several osteogenic factors regulate the development and differentiation of osteoblasts, such as bone morphogenetic proteins (BMPs), Runx2, and osterix [6]. BMPs belong to the transforming growth factor-β superfamily and play a key role in osteoblastic formation [7], while Runx2 and osterix are important osteogenic transcription factors that control bone mineralization and progression in mesenchymal stem cells and osteoblasts [8,9]. Enhancing Runx2 and osterix transcriptional activities apparently promotes osteoblastic differentiation and facilitates osteogenesis [5,9]. MicroRNAs (miRNAs) are small, endogenous non-coding RNAs (18–25 nucleotides in length) that have vital roles as modulators of several physiological and pathological processes [10,11]. miRNAs control gene expression at the post-transcriptional level by binding to the three prime untranslated region (3′-UTR) of mRNAs via complementary base pairing, leading to mRNA degradation or translation inhibition [12]. Several miRNAs have been found to modulate osteogenic functions of osteoblasts, including their development, proliferation, survival, and mineralization [12,13]. For instance, miR-6797-5p controls osteoblast differentiation by targeting Runx2 expression [14], while miR-96 regulates bone metabolism by modulating osterix expression [15]. These reports suggest that regulating miRNA expression is a critical tool for controlling osteoblastic function.

CCN3, otherwise known as the nephroblastoma overexpressed (NOV) protein, is a cysteine-rich protein that belongs to the CCN family and regulates several cellular functions such as cell proliferation, adhesion, and migration through its interactions with the extracellular matrix (ECM) [16]. CCN3 interacts with many integrin receptors, including integrins αvβ3, αvβ5, α2β1 and α5β1 [16]. The focal adhesion kinase (FAK), MAPK, PI3K, and Akt intracellular signaling pathways are commonly induced by CCN3 [17,18,19]. There is a lot of evidence that indicates that CCN3 regulates osteogenic factor expression and bone cell differentiation [20,21]. We have previously reported that CCN3 also increases BMP-4 production and bone mineralization in osteoblasts [17]. However, up until now, the role of CCN3 in regard to osteogenic transcription factors (Runx2 and osterix) has remained unclear. Here, we report that CCN3 enhances osteoblast differentiation and also promotes the expression of Runx2 and osterix in osteoblasts by inhibiting miR-608 expression via the FAK and Akt signaling pathways.

## 2. Results

### 2.1. CCN3 Promotes Osteoblast Differentiation

**Figure 1:**
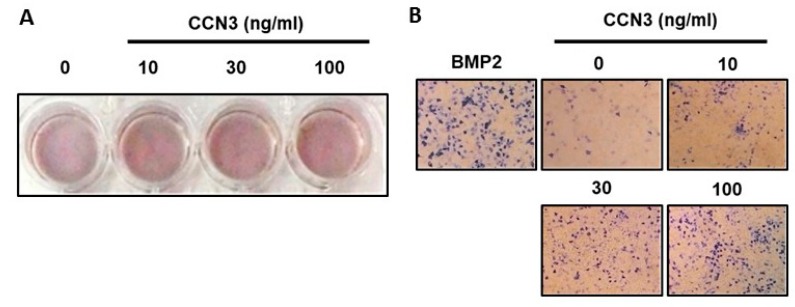
CCN3 enhances osteoblast differentiation. Osteoblasts were plated in 24-well plates and cultured in a medium containing vitamin C (50 μg/mL) and β-glycerophosphate (10 mM) for 2 weeks (**A**) or 2 days (**B**). The cells were also treated with CCN3. At the end of the experiment, the cultures were fixed and assessed by alizarin red-S (**A**) and alkaline phosphatase (ALP) staining (**B**).

In this study, we examined the role of CCN3 in osteoblast differentiation. After culturing osteoblasts in an osteoblastic differentiation medium (containing vitamin C 50 μg/mL and β-glycerophosphate 10 mM) for two weeks, alizarin red-S staining demonstrated that CCN3 promoted bone nodule synthesis (Figure 1A). We also found that CCN3 enhanced ALP expression (a marker for osteoblast differentiation) in a concentration-dependent manner, as according to ALP staining (BMP-2-enhanced ALP staining was used as a positive control) (Figure 1B). These results indicate that CCN3 enhances osteoblast differentiation.

### 2.2. CCN3 Enhances Runx2 and Osterix Expression in Osteoblasts

**Figure 2:**
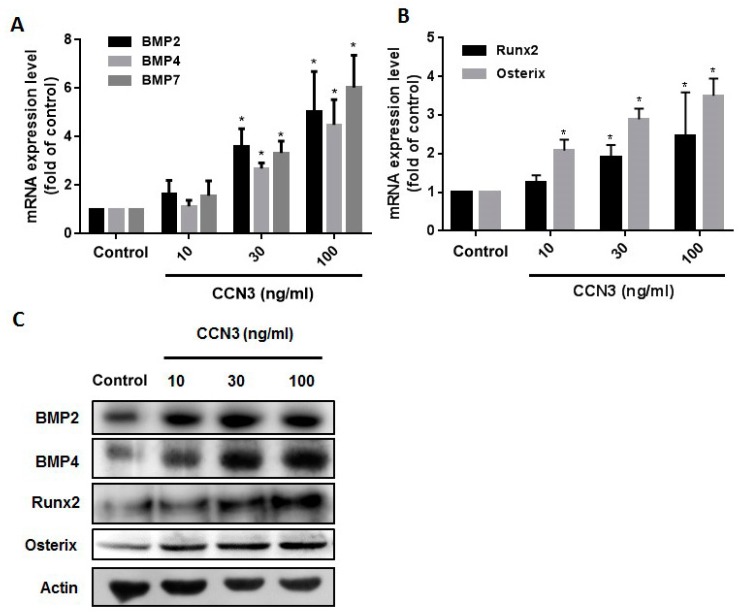
CCN3 enhances osteogenic factor expression in osteoblasts. (**A**,**B**) Osteoblasts were treated with CCN3 (10–100 ng/mL) for 24 h; mRNA and protein expression were examined by qPCR (**A**,**B**) and Western blot assay (**C**), respectively. Results are expressed as the mean ± S.E. * *p* < 0.05 as compared with the control group.

Treatment of osteoblasts with CCN3 increased mRNA and protein expression of BMP-2, BMP-4, and BMP-7, in a concentration-dependent manner (Figure 2A,C). CCN3 also augmented Runx2 and osterix expression in osteoblasts (Figure 2B,C). These results suggest that CCN3 promotes the expression of osteogenic factors in osteoblasts.

### 2.3. CCN3 Increases Runx2 and Osterix Expression via the Suppression of miR-608

**Figure 3:**
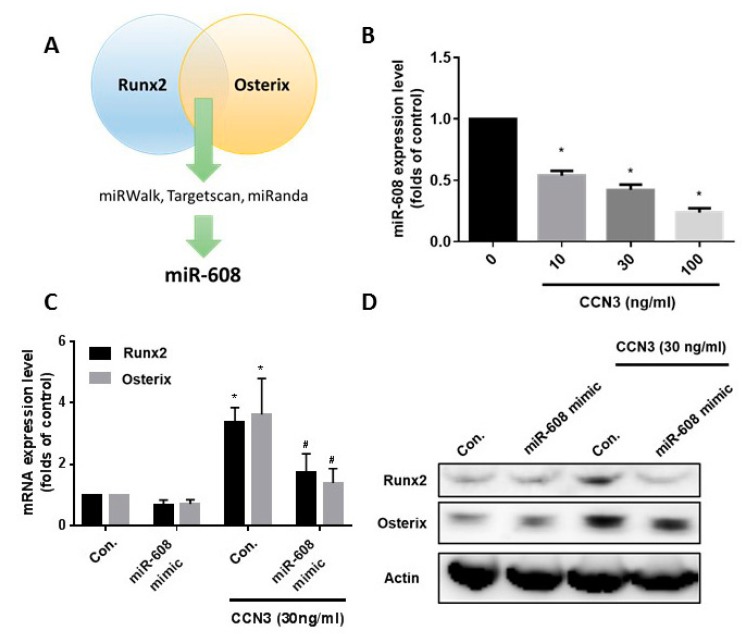
CCN3-induced suppression of miR-608 enhances Runx2 and osterix expression. (**A**) Open-source software (miRWalk, miRanda, and Targetscan) was utilized to identify microRNAs (miRNAs) that could possibly interfere with Runx2 and osterix transcription. (**B**) Osteoblasts were incubated with CCN3 for 24 h and miR-608 expression levels were examined by qPCR assay. Osteoblasts were transfected with miR-608 mimic and subsequently stimulated with CCN3. mRNA and protein expression of Runx2 and osterix were examined by qPCR (**C**) and Western blot assay (**D**), respectively. Results are expressed as the mean ± S.E. * *p* < 0.05 as compared with the control group; # *p* < 0.05 as compared with the CCN3-treated group.

**Figure 4:**
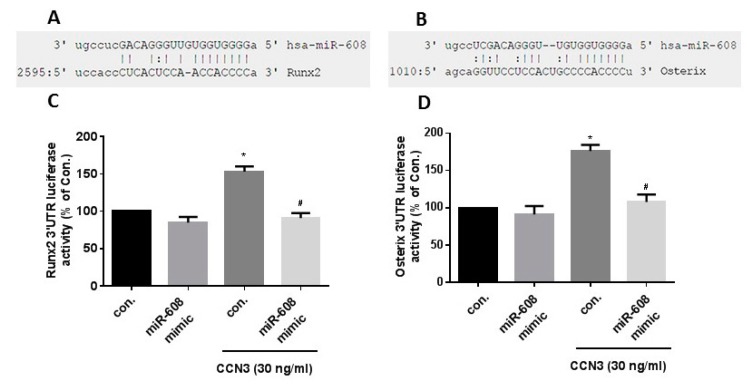
The binding of miR-608 to Runx2 and osterix three prime untranslated region (3′-UTRs) mitigated CCN3-induced increases in Runx2 and osterix expression. (**A**,**B**) Diagram of the miR-608 binding sites in the Runx2 and osterix 3′-UTRs. (**C**,**D**) Osteoblasts were transfected with the Runx2 or osterix 3′-UTR plasmids, with or without miR-608 mimic, then stimulated with CCN3. Luciferase promoter activity was expressed as relative luciferase activity. Results are expressed as the mean ± S.E. * *p* < 0.05 as compared with the control group; # *p* < 0.05 as compared with the CCN3-treated group.

The online miRWalk, miRanda, and TargetScan databases revealed that the 3′-UTRs of Runx2 and osterix mRNAs harbor potential binding sites for only miR-608 (Figure 3A). We found that stimulation of CCN3 with osteoblasts concentration-dependently inhibited miR-608 expression (Figure 3B). To examine the role of miR-608 in Runx2 and osterix expression, the miR-608 mimic was used. Transfection with miR-608 mimic antagonized CCN3-increased Runx2 and osterix expression (Figure 3C,D). To examine whether miR-608 regulates the 3′-UTRs of Runx2 and osterix, we constructed luciferase reporter vectors harboring either the 3′-UTR of Runx2 mRNA or the 3′-UTR of osterix mRNA (Figure 4A,B) and transfected them into osteoblasts. CCN3 increased luciferase activity in the Runx2 and osterix plasmids (Figure 4C,D). Transfection with miR-608 mimic reduced CCN3-promoted Runx2 and osterix luciferase activity (Figure 4C,D), suggesting that CCN3 induces Runx2 and osterix activation via the inhibition of miR-608 expression.

### 2.4. CCN3 Stimulates Runx2 and Osterix Expression by Inhibiting miR-608 through the FAK and Akt Signaling Pathways

**Figure 5:**
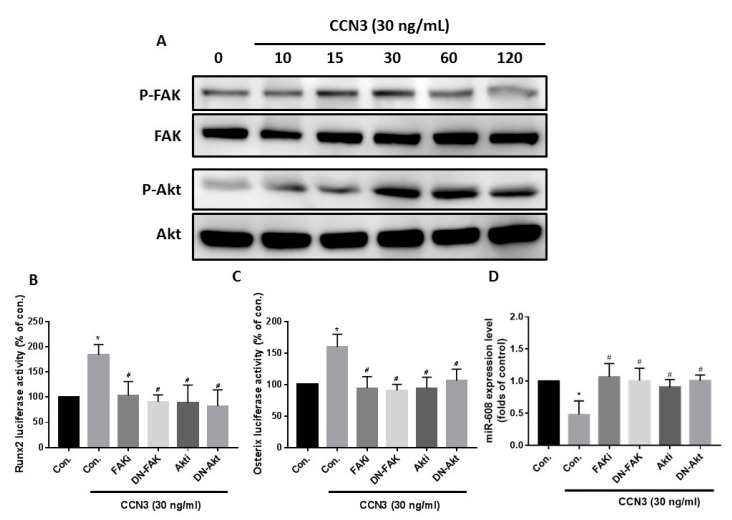
CCN3 suppressed miR-608 expression through the FAK and Akt signaling pathways. (**A**) Osteoblasts were treated with CCN3 (30 ng/mL) for the indicated time intervals; FAK and Akt phosphorylation were examined by Western blot assay. Osteoblasts were pretreated with FAK and Akt inhibitors or transfected with FAK and Akt mutants and subsequently stimulated with CCN3. Runx2 and osterix luciferase activity and miR-608 expression were examined by luciferase assay (**B**,**C**) and qPCR (**D**), respectively. Results are expressed as the mean ± S.E. * *p* < 0.05 as compared with the control group; # *p* < 0.05 as compared with the CCN3-treated group.

Stimulation of osteoblasts with CCN3 led to a time-dependent increase in phosphorylation of FAK and Akt, as shown by the Western blot assay (Figure 5A). To validate the role of FAK and Akt in CCN3-enhanced Runx2 and osterix expression, osteoblasts were pretreated with FAK and Akt inhibitors or transfected with FAK and Akt mutants. A luciferase activity assay confirmed significant inhibition of CCN3-enhanced Runx2 and osterix luciferase activities, as shown in Figure 5B,C. In addition, FAK and Akt inhibitors or mutants reversed CCN3-inhibited miR-608 expression (Figure 5D). These data suggest that miR-608 directly suppresses Runx2 and osterix gene transcription via binding to the 3′-UTR region of the human Runx2 and osterix gene promoter, and that miR-608 expression is negatively regulated by FAK and Akt phosphorylation induced by upstream CCN3 signaling.

## 3. Discussion

The proliferation and differentiation of osteoblasts is regulated by different endogenous factors [22]. CCN3 promotes BMP-4-dependent bone mineralization in osteoblasts [17], although the effects of CCN3 upon two important osteogenic transcription factors, Runx2 and osterix, are largely unknown. In the current study, we found that CCN3 promotes BMP expression and the differentiation of osteoblasts and that CCN3 stimulation increases Runx2 and osterix expression by inhibiting miR-608 expression via the FAK and Akt signaling pathways. We also found that CCN3 increases levels of BMP, Runx2, and osterix expression in osteoblasts. Conversely, BMP has been implicated in the enhancement of Runx2 and osterix activity [23]. Whether CCN3 promotes Runx2 and osterix activation via a BMP-dependent effect needs further examination.

Controversy surrounds the contention that CCN3 modulates bone cell function. Rydziel et al. indicated that CCN3 inhibits osteoblastogenesis and causes osteopenia in vivo [21] and another research group also found that CCN3 inhibits osteoblast differentiation through the inhibition of BMP-2 expression in osteoblast precursor cells [20], while a higher dosage of CCN3 (600 ng/mL) inhibits the differentiation of primary bone marrow cells [24]. However, we have previously reported that CCN3 enhances BMP-4-dependent bone mineralization [17]. The results of this current study support our previous finding that CCN3 enhances osteoblast differentiation. In addition, we found that CCN3 stimulation upregulated levels of osteogenic factors, including BMP-2, BMP-4, BMP-7, Runx2, and osterix. The opposite effect may therefore depend on the working concentration of CCN3, as we found that a lower dosage of CCN3 (30 ng/mL) increases osteoblast differentiation, whereas a higher CCN3 dosage (600 ng/mL) reportedly inhibits this phenomenon. Otherwise, most inhibitory effects have been found through the overexpression of CCN3. However, this effect may depend on the route of treatment, as in this study, we applied exogenous recombinant CCN3 to osteoblasts. Several different cells have been used in bone function assays, including primary bone marrow cells, stromal cells, and osteoblastic cells. Thus, the opposite effect may depend on the concentration, timing of the stimulation, route of treatment, and type of cell.

miRNAs are small, non-coding RNA fragments that suppress the translation or induce the degradation of target mRNAs [25]. A multitude of miRNAs are known to be involved in bone-related disorders [26,27,28]. We utilized open-source software (miRWalk, miRanda, and TargetScan) to evaluate candidate miRNAs that may interfere with the transcription of Runx2 and osterix. Among the selected miRNAs, only miR-608 regulated both Runx2 and osterix transcriptional activity. We have shown that transfecting osteoblasts with miR-608 mimic mitigates CCN3-stimulated Runx2 and osterix expression. These findings underscore the importance of miR-608 in CCN3-stimulated Runx2 and osterix expression. BMP family proteins play a critical role in bone formation and differentiation [29]. However, we have not found any reports in the literature regarding miR-608-induced regulation of BMPs. Whether BMPs also modulate miR-608-dependent bone formation needs further examination. miR-608 activity has been mentioned in several cancers; for example, miR-608 regulates apoptosis in lung adenocarcinoma [30] and the miR-608 rs4919510 C>G polymorphism is associated with a significantly lower risk of breast cancer [31]. However, the effects of miR-608 in bone cells are not yet quantified. Whether miR-608 also controls other bone cell functions needs further investigation.

Activation of the FAK pathway regulates osteoblast adhesion and differentiation [32,33]. Akt activation is also implicated in osteoblastic functions [34,35]. In this investigation, CCN3 augmented the phosphorylation of FAK and Akt. In addition, FAK, Akt inhibitors, and their associated mutants all abolished CCN3-induced elevations in Runx2 and osterix expression, indicating that FAK and Akt signaling mediates the effects of CCN3. These inhibitors and their mutants also reversed CCN3-inhibited expression of miR-608, suggesting that the FAK/Akt pathway acts as an upstream molecule of miR-608. These findings provide evidence showing that CCN3 enhances the expression of transcription factors Runx2 and osterix by inhibiting miR-608 expression via the FAK and Akt signaling cascades. We previously reported that CCN3 regulates BMP-4 production through the MAPK pathway [17]. Treatment of osteoblasts with ERK, p38, and JNK inhibitors reversed CCN3-inhibted miR-608 expression (data not shown), suggesting that the MAPK pathway is also involved in CCN3-induced miR-608 suppression.

## 4. Materials and Methods

### 4.1. Materials

We obtained recombinant human CCN3 and BMP-2 from PeproTech (Rocky Hill, NJ, USA) and purchased BMP-2, BMP-4, Runx2, and osterix antibody from Abnova (Taipei, Taiwan). Antibodies against p-FAK, FAK, p-Akt, Akt, and β-actin were purchased from Santa Cruz (Santa Cruz, CA, USA) and cell culture supplements from Invitrogen (Carlsbad, CA, USA). The Dual-Luciferase^®^ Reporter Assay System was purchased from Promega (Madison, WI, USA). Quantitative polymerase chain reaction (qPCR) primers and probes, as well as the Taqman^®^ one-step PCR Master Mix, were supplied by Applied Biosystems (Foster City, CA, USA). All other chemicals not mentioned above were supplied by Sigma-Aldrich (St Louis, MO, USA). The FAK dominant-negative (DN) mutant was a gift from Dr. J. A. Girault (Institut du Fer á Moulin, Paris, France). The Akt DN mutant was gifted by Dr. W. M. Fu (National Taiwan University, Taipei, Taiwan).

### 4.2. Cell Culture

The osteoblastic cell line MC3T3-E1 was purchased from American Type Culture Collection (Manassas, VA, USA). Cells were cultured in culture media containing alpha-minimal essential medium (α-MEM) supplemented with streptomycin (100 μg/mL), penicillin (100 U/mL), HEPES (20 mM), glutamine (2 mM), and 10% fetal bovine serum (FBS), then maintained at 37 °C in an atmosphere of humidified air with 5% CO_2_.

### 4.3. Measurement of Osteoblast Differentiation

Cells were cultured in a medium containing vitamin C (50 μg/mL), β-glycerophosphate (10 mM), and CCN3 (10–30 ng/mL) for 2 weeks. Cells were fixed in ice-cold 75% (*v*/*v*) ethanol for 30 min and the calcium deposition was determined using 40 mM alizarin red-S staining (pH 4.2) [36].

For alkaline phosphatase (ALP) staining, the cells were fixed with acetone for 30 s then stained with ALP staining reagent (6 mg naphthol AS phosphate, 0.1 mL *N*,*N*-dimethylformamide, and 20 mg Fast Blue BB salt in 20 mL of 0.5 M Tris buffer; pH 10.2). Images were photographed using a microscope (Nikon, Kanagawa, Japan) [37].

### 4.4. Western Blot Analysis

Cell lysates were prepared by RIPA buffer containing a protease inhibitor cocktail (Sigma-Aldrich; St Louis, MO, USA); extracted proteins were analyzed by sodium dodecyl sulfate polyacrylamide gel electrophoresis and transferred to Immobilon^®^ polyvinylidene difluoride membranes. The blots were treated with 4% bovine serum albumin (BSA) for 1 h and then with primary antibodies (1:3000) for 1 h. The membranes were treated with peroxidase-conjugated secondary antibody (1:3000) for 1 h. Finally, the bands were visualized using ImageQuant™ LAS 4000 (GE Healthcare, Little Chalfont, UK) [38,39].

### 4.5. Quantitative Real-Time PCR

Total cDNA (100 ng) was mixed with Taqman^®^ primers and probes as well as PCR Master Mix. The StepOnePlus™ system was used in the quantitative RT-PCR assays. BMP-2 (F) GGGACCCGCTGTCTTCTAGT, BMP-2 (R) TCAACTCAAATTCGCTGAGGAC; BMP-4 (F) TTCCTGGTAACCGAATGCTGA, BMP-4 (R) CCTGAATCTCGGCGACTTTTT; BMP-7 (F) ACGGACAGGGCTTCTCCTAC, BMP-7 (R) ATGGTGGTATCGAGGGTGGAA; Runx2 (F) CCAACCGAGTCATTTAAGGCT, Runx2 (R) GCTCACGTCGCTCATCTTG; osterix (F) ATGGCGTCCTCTCTGCTTG, osterix (R) TGAAAGGTCAGCGTATGGCTT. For the detection of miRNAs, reverse transcription was performed using Mir-X™ miRNA First-Strand Synthesis and the SYBR^®^ RT-PCR kit. qPCR analysis was carried out according to an established protocol [40,41].

### 4.6. Plasmid Construct and Reporter Assay

We obtained Runx2 and osterix 3′-UTR DNA fragments from Invitrogen (Carlsbad, CA, USA) and subcloned them into the pmirGLO-control luciferase reporter vector (Promega, Madison, WI, USA). Luciferase activity was assayed using the method as described in our previous research [42,43].

### 4.7. Statistics

All values are presented as the mean ± the standard error (S.E.). All differences between the experimental groups and controls were assessed for significance using the Student’s *t*-test. Between-group differences were considered to be significant if the *p* value was <0.05.

## 5. Conclusions

**Figure 6:**
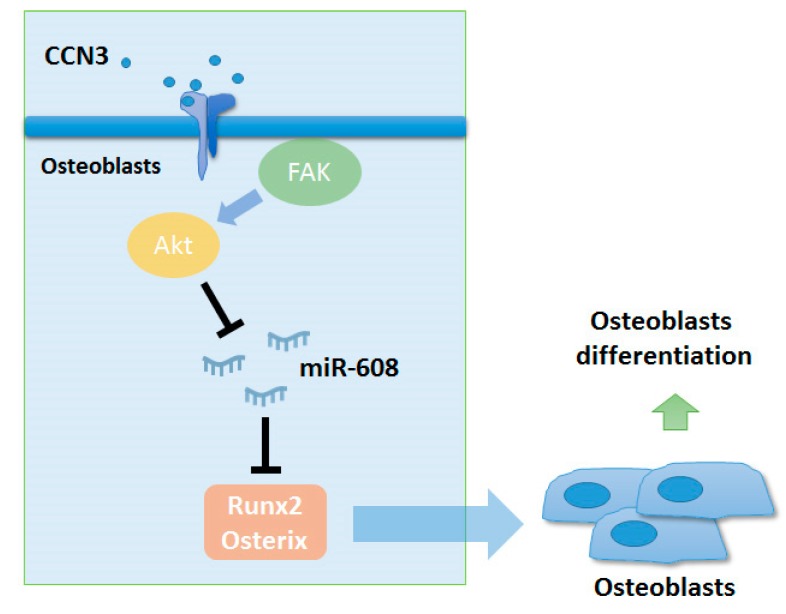
Schematic diagram summarizes the mechanism whereby CCN3 promotes Runx2 and osterix expression in osteoblasts. CCN3 promotes the expression of osteogenic transcriptional factors Runx2 and osterix in osteoblasts by downregulating miR-608 through the focal adhesion kinase (FAK) and Akt signaling pathway.

In summary, our study shows that the binding of CCN3 in osteoblasts triggers the phosphorylation of FAK and Akt, contributing to the decline in miR-608 synthesis. The decrease in miR-608 expression enhances the synthesis of osteogenic transcription factors Runx2 and osterix (Figure 6). These results may improve understanding of the role played by CCN3 in osteoblast differentiation.
